# Supplementary material for: The Effects of Moxifloxacin and Gemifloxacin on the ECG Morphology in Healthy Volunteers: A Phase 1 Randomized Clinical Trial
Source: Diagnostics (Basel). 2023 Mar 24;13(7):1234. doi: 10.3390/diagnostics13071234 (PMC10092949; doi:10.3390/diagnostics13071234)
Supplement: Supplementary file 1 [file diagnostics-13-01234-s001.zip › diagnostics-2271127-supplementary.pdf]

Table S1. PROFOMA FOR REPORTING ADRs DURING CLINICAL TRIAL PERIOD

Volunteer Name			Age	BP	Dated:	Pulse rate	
**Sex**	**M**	**F**	**Contact No**				
**Marital status**	**Married**		**Unmarried**			**Weight**	
**Address**							
**Family History**							

**Fluroquinolones used**			**Daily Dose**	**Dose duration**	**Yes/NO**
1	*Moxifloxacin*		400 mg	5 days	
2	*Gemifloxacin*		325 mg	5 days	

Adverse/Side Effects and Non-Compliance:

**S.no.1**	***Moxifloxacin***	**Compliance (Yes/No)**
**S.no .2**	***Gemifloxacin***	**Compliance (Yes/No)**
